# Human and remote sensing data to investigate the frontiers of urbanization in the south of Mexico City

**DOI:** 10.1016/j.dib.2016.12.049

**Published:** 2016-12-29

**Authors:** Juan Miguel Rodriguez Lopez, Katharina Heider, Jürgen Scheffran

**Affiliations:** a Center for Sustainable University and CliSAP, University of Hamburg, Grindelberg 5, 20144 Hamburg, Germany; b Research Group Climate Change and Security (CLISEC), Institute of Geography and CliSAP/CEN, University of Hamburg, Grindelberg 5, 20144 Hamburg, Germany

## Abstract

The data presented here were originally collected for the article “Frontiers of Urbanization: Identifying and Explaining Urbanization Hot Spots in the South of Mexico City Using Human and Remote Sensing” (Rodriguez et al. 2017) [4]. They were divided into three databases (remote sensing, human sensing, and census information), using a multi-method approach with the goal of analyzing the impact of urbanization on protected areas in southern Mexico City. The remote sensing database was prepared as a result of a semi-automatic classification, dividing the land cover data into urban and non-urban classes. The second data set details an alternative view of the phenomena of urbanization by concentrating on illegal settlements in the conservation zone. It was based on voluntary complaints about environmental and land use offences filed at the Procuraduria Ambiental y del Ordenamiento Territorial del Distrito Federal (PAOT), which is a governmental entity responsible for reviewing and processing grievances on five basic topics: illegal land use, deterioration of green areas, waste, noise/vibrations, and animals. Anyone can file a PAOT complaint by phone, electronically, or in person. The complaint ends with a resolution, act of conciliation, or recommendation for action by other actors, such as the police or health office. The third data about unemployment was extracted from Mexico׳s National Census 2010 database available via public access.

**Specifications Table**

**Table:** 

Subject area	Sustainability, urbanization, geography
More specific subject area	Sustainable land use, Volunteered Geographic Information (VGI), GIS
Type of data	Satellite images, VGI, python code, census data, ArcGIS toolbox
How data was acquired	Underlying RapidEye data from the German Aerospace Center was obtained through funding by the German Federal Ministry of Economy and Energy. VGI was obtained from the PAOT (on September 3, 2015) and 2010 census data was downloaded from Mexico׳s National Census Bureau, (INEGI, 2010), both open access. For further analysis, ArcGIS 10.3 was used.
Data format	TIF (analyzed), SHP, DBF, TBX (ArcGIS toolbox), python file.
Experimental factors	The analysis based on a grid obtained through optimal value of autocorrelation using ArcGIS 10.3, the optimized hot spot analysis tool.
Experimental features	Combination of remote and human sensing (steps 1 and 2 of the graphical abstract [4]) with census information in a framework of optimized hot spot analysis.
Data source location	South of Mexico City
Data accessibility	Data are included in this paper

**Value of the Data**

• As the frontiers of urbanization are spaces with a high potential for conflict, these new data and combination of data types present a methodology with the capacity for replication of studies in many cities.
• A combination of human (VGI) and remote sensing is relevant for a more reliable and validated analysis of urban growth.
• Due to the decreasing costs of satellite imagery and the increasing availability of VGI, this type of analysis has been proven to be cost effective for future urban growth research.
• Other socio-economic variables can be easily integrated into the analysis for further investigation.

## 1 Data

**Fig. 1:**
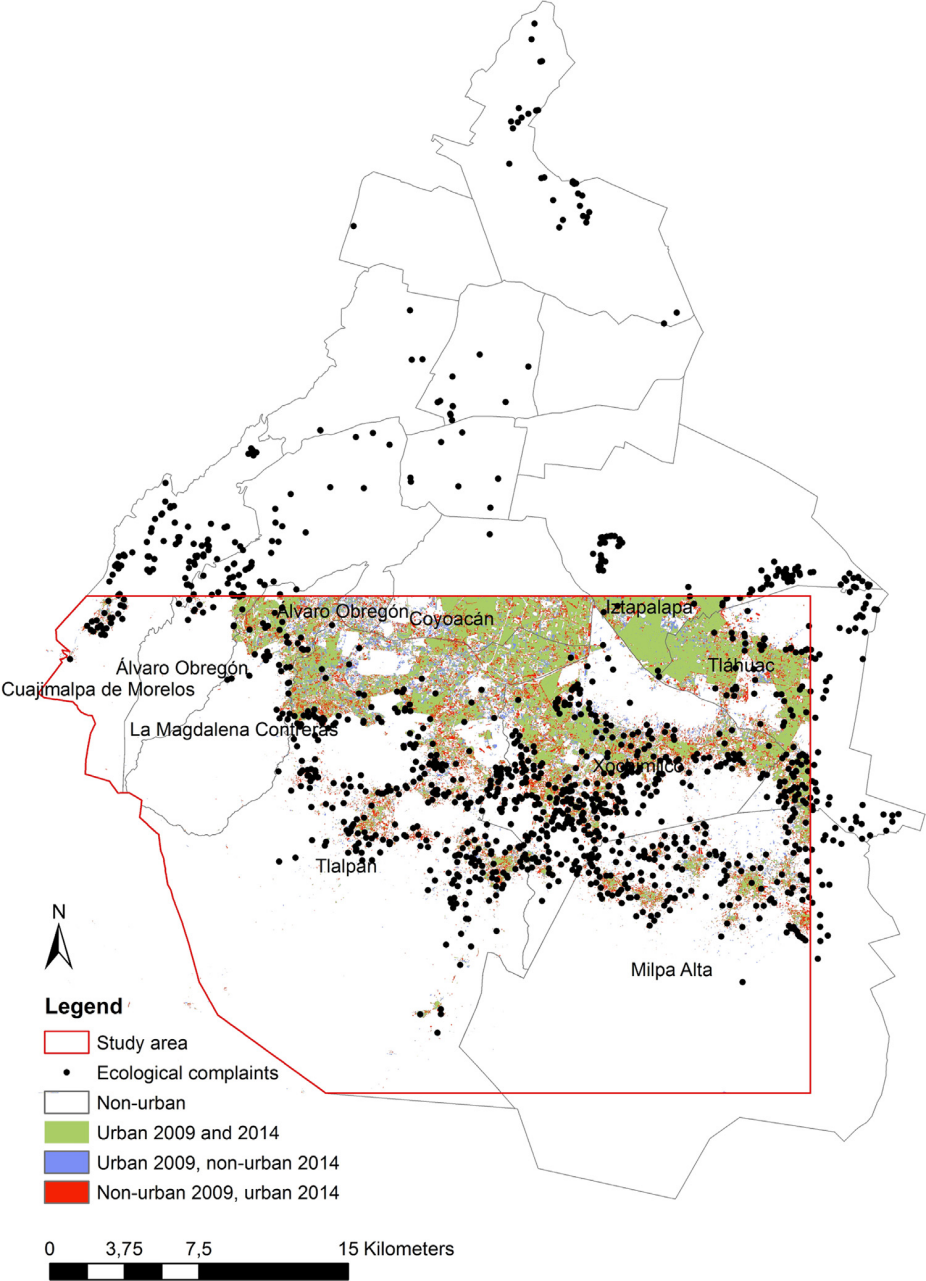
Data for the southern part of the Federal District in Mexico City: urban change, calculated from 5 m remote sensing imagery of the RapidEye Science Archive (RESA) program, and ecological complaints (human sensing) from PAOT.

For the urban change data, two tiles (IDs 1447913 and 1447914) of RapidEye 3A level products (5 m per pixel) covering the south of Mexico City in November 2009 and August 2014 were classified using E-cognition [5], which is a standard software for land use change analysis. Afterwards, we conducted change detection. The given urban change data contain four classes: 0=non-urban in 2009 and 2014; 1=urban in 2009 and 2014; 2=urban in 2009 but non-urban in 2014; and 3=non-urban in 2009 but urban in 2014. The ecological complaints were reported between 2002 and 2013. The data set includes 971 complaints concerning violations of the status of the conservation area (see Fig. 1 and related paper [4] for an explanation of “conservation area”). The smallest available basic geostatistical area (AGEB) contained a shape file with population data already included in the attributed table and a table containing economic characteristics for the Federal District of Mexico City. Important abbreviations for our analysis in the data set include: pop1=total population and eco25_R=unemployment rate (original name of the variables). A shape file of the study area is included (see Fig. 1) as well as an ArcGIS toolbox containing the ModelBuilder for the analysis of the urban change data. Fig. 3 illustrates the model. The same workflow was added as python code.

## 2 Experimental design, materials, and methods

**Fig. 2:**
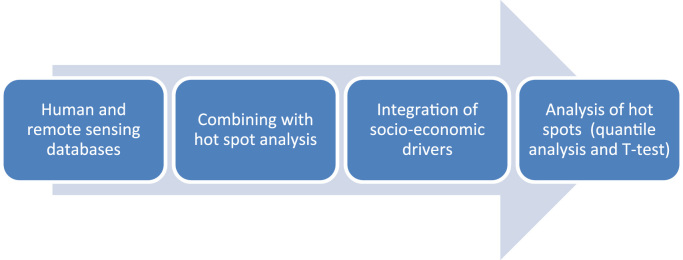
Overview of methods, combining human and remote sensing.

We investigated the growth of illegal settlements on the frontiers of urbanization in Mexico City using a combination of human observation (VGI) and remote sensing (see Fig. 2). A graphical abstract can be found in the associated publication [4]. In the following analysis, statistically significant urbanization hot spots were identified and investigated based on human and remote sensing. Integrating socio-economic drivers from the census [3], a statistical analysis of the causes of the growth in urbanization follows in future publications.

In order to compare data from both human and remote sensing, it was necessary to work with the same data type. Vector data were favorable for most parts of the analysis. As the ecological complaints were already available as point data, the urbanization class from the change detection based on RapidEye imagery had to be extracted and converted to points. Each pixel classified as non-urban in 2009 and urban in 2014 was converted into one point. In the next step, we conducted the optimized hot spot analysis using ArcGIS 10.3. Within the analysis, statistically significant hot and cold spots were identified with the Getis-Ord Gi* statistic [1]. The analysis of the cluster was based on the distance between each point (i.e., every pixel in the remote sensing data or every complaint in the complaints database) and the nearest neighbor. In this analysis, we compared the observation points and a random sample, obtaining a statistically significant classification of areas in various forms of clustering: hot and cold spots [2]. Polygons were the spatial units of aggregation. The cell size of the remote sensing data was adapted to the cell size of the complaints data set, creating a grid with a target resolution of 561 m, before the optimized hot spot analysis for the remote sensing data was conducted. In the complaints data set, 561 m generated the best results for the autocorrelation test of the automatic identification of hot and cold spots. While significant hot spots received a positive value, significant cold spots received a negative one. The significance was 90% for values of +/−1, 95% for values of +/−2, and 99% for values of +/−3. A lower level of significance was assigned with a value of 0.

**Fig. 3:**
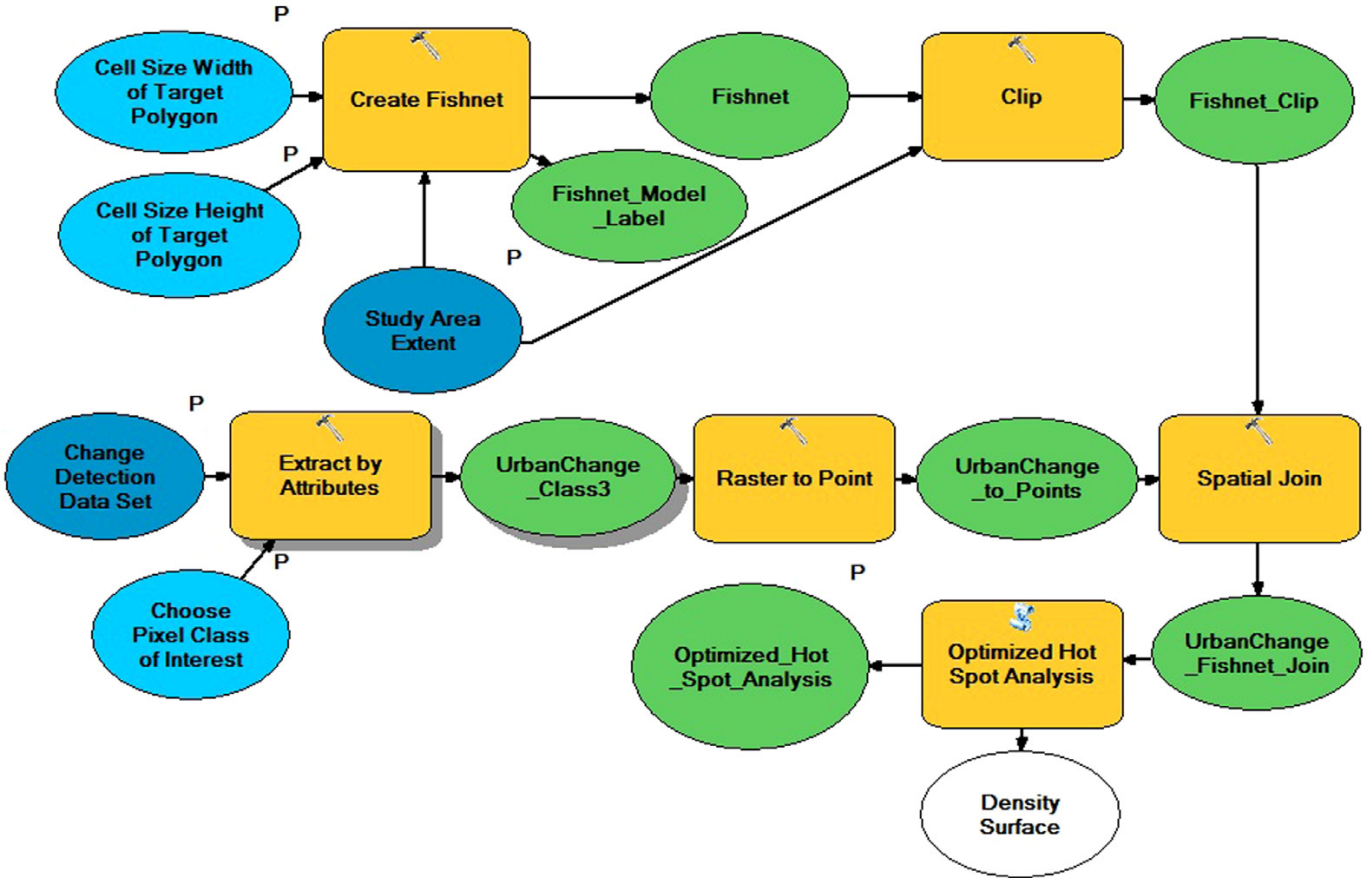
ModelBuilder representing the analysis of urban change data based on 5 m remote sensing imagery (RESA program) to identify statistically significant hot spots.

**Fig. 4:**
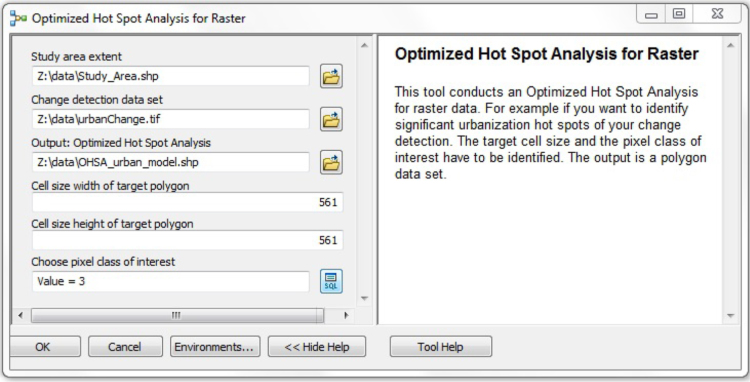
Interface of the ModelBuilder included in this paper (note: for application, all file paths need to be adapted).

An optimized hot spot analysis was executed for the ecological complaints and for the remote sensing data. Fig. 3 shows an image of the ModelBuilder using ArcGIS 10.3, as well as the steps for the analysis of remote sensing data, which allows for easy replication. Data for the analysis and an ArcGIS toolbox containing the ModelBuilder are included in this paper. The ModelBuilder shows a standard representation of the analysis produced by ArcGIS. P indicates a model parameter, the blue and green ovals represent input and output variables, and yellow is a tool element; for a detailed description of the ArcGIS ModelBuilder׳s elements see the ESRI tutorial.^1^ For the application, we added the data and toolbox to ArcGIS. Fig. 4 shows the interface of the ModelBuilder. To identify overlapping areas, we overlaid the hot spots maps. The intersect tool made it possible to identify the precise number of these overlapping cells. To calculate how much human sensing explained the observed urbanization by remote sensing, we analyzed the number of cells belonging to hot spots with 99% confidence for both data sets [4].

However, a key limitation of this approach using ArcGIS is the cost associated with software licensing. The Python script allows for a more transparent presentation of the analysis with this software. A file with the detailed code has been added here. A particular note is that the names of the folders and paths should be adjusted to run this file. We recommend using a file geodatabase for data storage.

**Fig. 5:**
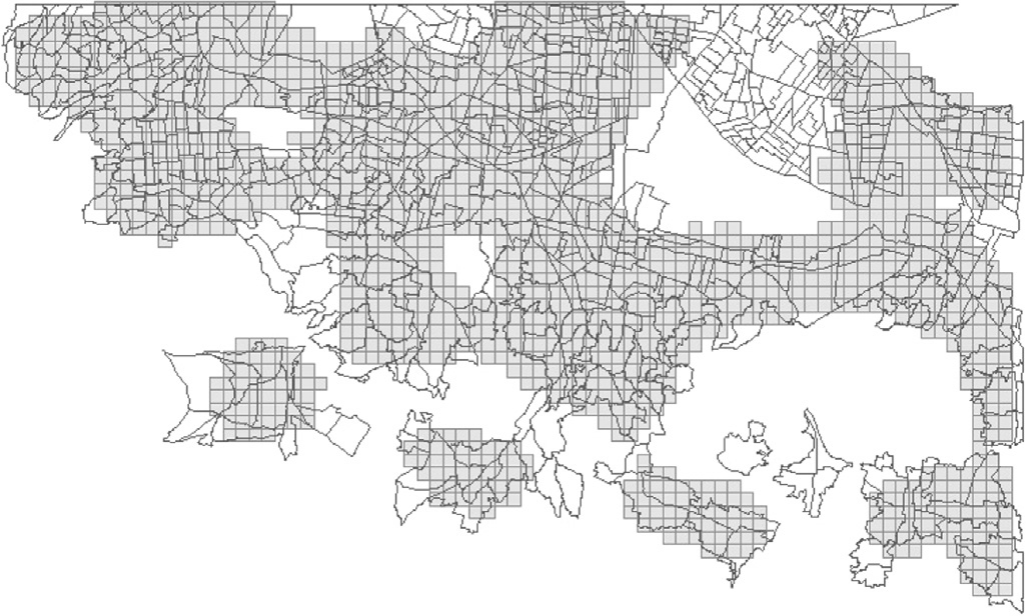
Size distribution of AGEB and fishnet in the Federal District (overlay).

For the integration of socio-economic drivers, population and unemployment data from Mexico City, AGEB data was used from the National Census 2010 [3]. The AGEB areas differed greatly in size. While the smallest area was 0.006 km^2^, the biggest area was 7.25 km^2^. The mean size was 0.4 km^2^ with a standard deviation of 0.504 km^2^. To minimize the loss of accuracy and ensure the consistency of our analysis, we chose a grid size of 0.143 km^2^. To transfer the data to the target grid (378 m), we made a spatial join using the mean value as the merge rule and intersect as the match option. Although the fishnet was smaller, both the fishnet and AGEBs were comparable because the difference was acceptable, with a loss of accuracy. Both layers are shown in Fig. 5 to estimate the loss of accuracy.

## Footnotes

1 Esri tutorial of the ModelBuilder can be found here: http://help.arcgis.com/en/arcgisdesktop/10.0/pdf/creating-tools-in-modelbuilder-tutorial.pdf (Accessed 20.12.2016).
