# Supplementary material for: Human and remote sensing data to investigate the frontiers of urbanization in the south of Mexico City
Source: Data Brief. 2016 Dec 29;11:5–11. doi: 10.1016/j.dib.2016.12.049 (PMC5227549; doi:10.1016/j.dib.2016.12.049)
Supplement: Supplementary file 2 — Supplementary material [file mmc2.pdf]

Data article

Title: ***Human and Remote Sensing Data to Investigate the Frontiers of Urbanization in the South of Mexico City***

Authors: Juan Miguel Rodriguez Lopez(a), Katharina Heider(b), Jürgen Scheffran(b)

Affiliations:

~~aCorresponding~~(a) Corresponding author. Center for Sustainable University and CliSAP, University of Hamburg, Grindelberg 5, 20144 Hamburg, Germany. E-mail: miguel.rodriguez@uni-hamburg.de

~~bResearch~~(b) Research Group Climate Change and Security (CLISEC), Institute of Geography and CliSAP/CEN, University of Hamburg, Grindelberg 5, 20144 Hamburg, ~~-~~Germany. E-~~Mail~~mail: info@katharina-heider.de; juergen.scheffran@uni-hamburg.de.

Contact ~~email~~e-mail: miguel.rodriguez@uni-hamburg.de

Abstract

The data presented ~~data is~~here were originally collected for the article "Frontiers of Urbanization: Identifying and Explaining Urbanization Hot Spots in the South of Mexico City Using Human and Remote Sensing"[4]. They were divided into three databases (remote sensing, human sensing, and census information~~) applying~~), using a multi-methods approach~~. ~~ with the goal of analyzing the impact of urbanization on protected areas in southern Mexico City. The remote sensing database ~~is the~~was prepared as a result of a semi-automatic classification, ~~which was carried out by preliminary work (Rodriguez Lopez et al. 2015). The~~ dividing the land cover data into urban and non-urban classes ~~included in this analysis are: urban, forest, non-forest vegetation (grassland and crops), bare soil and water. Two classes (urban and not urban) sum up the final set of land cover classes, considering that the main goal of this study is to analyze the impact of urbanization on protected areas. Although the forest land cover is important to estimate the impact of urbanization on protected areas, ultimately, the effect of urban growth needs to be assessed based on the legal coverage of protected areas.~~. The second data set details an alternative view ~~on~~of the phenomena of urbanization by concentrating on illegal settlements in the conservation zone. It ~~is~~was based on ~~volunteered~~voluntary complaints about environmental and land use offences~~. These complaints were~~ filed at the ~~"~~Procuraduria Ambiental y del Ordenamiento Territorial del Distrito Federal~~"~~ (PAOT), which is a governmental entity responsible for reviewing and processing~~. The human sensing database was obtained from the PAOT on September 3, 2015, but part of this database is available online on the PAOT website (www.paot.org.mx). Figure 1 displays both databases in the research area.~~

~~The PAOT collects complaints~~ grievances on five basic topics: illegal land use, deterioration of green areas, waste, noise~~ / ~~/vibrations, and animals. Anyone can ~~start the procedure to~~ file a PAOT complaint by phone, electronically, or in person. ~~As for procedures, the PAOT should first decide on the legal character of the complaint, admitting it within 13 working days. After this admission, the PAOT~~

~~generates a preliminary report within 30 working days.~~ The complaint ends with ~~the~~a resolution, act of conciliation~~ act~~, or recommendation for action by other actors, such as the police or ~~the~~ health office. The third data ~~set is~~about unemployment was extracted from Mexico's National Census 2010 database ~~(National Census Bureau in Mexico, INEGI, 2010) and it is also of~~available via public access.

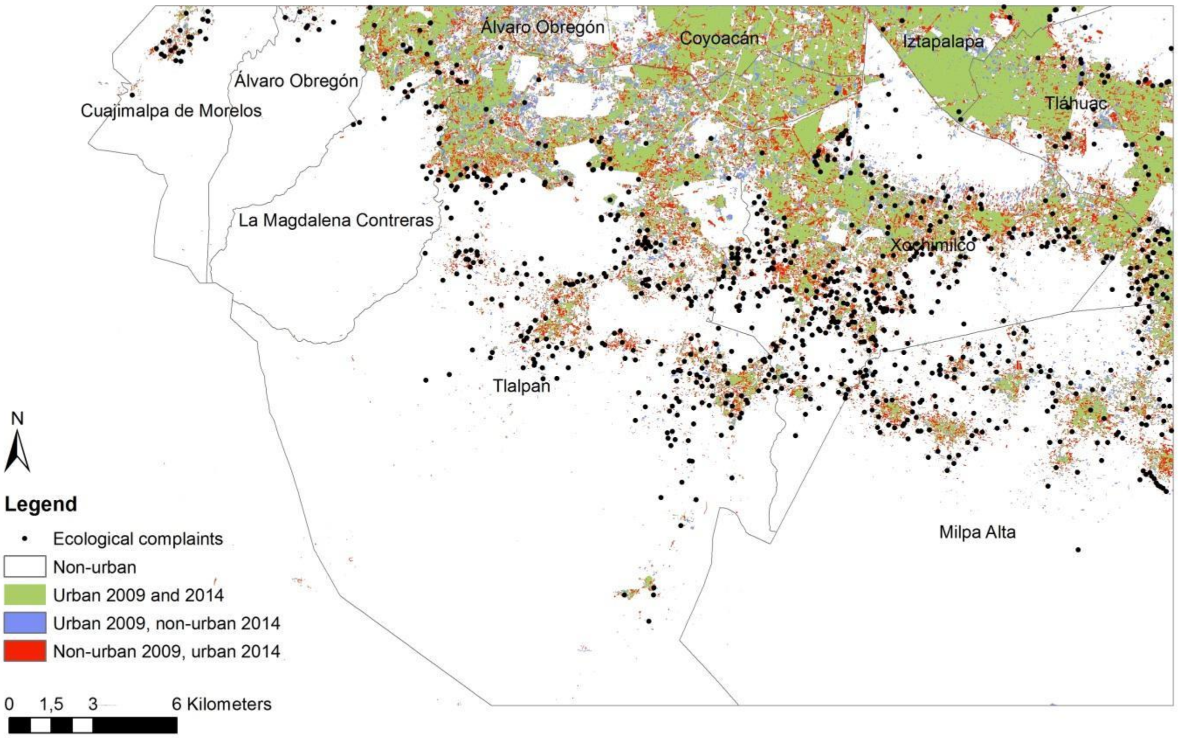

~~[Figure 1 Location of data in the southern part of the Federal District in Mexico City: urban change, calculated from 5m remote sensing imagery from the RESA program, and ecological complaints (human sensing) from PAOT][around here]~~

Specifications Table

Subject area	Sustainability, ~~Urbanization, Geography~~urbanization, geography
More specific subject area	Sustainable land use, Volunteered Geographic Information (VGI), GIS

Type of data	Satellite ~~image~~images, VGI, python code, census data, ~~-~~ArcGIS toolbox
How data was acquired	Underlying RapidEye data ~~was contributed on behalf of~~from the German Aerospace Center was obtained through funding by the German Federal Ministry of Economy and Energy. VGI was obtained from the PAOT (on September 3, 2015~~),~~) and 2010 census data ~~2010~~ was downloaded from Mexico's National Census Bureau ~~in Mexico,~~ , (INEGI, 2010 ~~(~~), both open access~~).~~<br>. For further analysis, ArcGIS 10.3 was used.
Data format	~~Tif~~TIF (analyzed), ~~shape, dbf, tbx~~SHP, DBF, TBX (ArcGIS toolbox), python file
Experimental factors	The analysis ~~is~~ based on a grid obtained through optimal value of autocorrelation using ArcGIS 10.3, the optimized hot spot analysis tool ~~of ArcGIS 10.3~~.
Experimental features	Combination of remote and human sensing (steps 1 and 2 of the graphical abstract~~, see Rodriguez Lopez et al. 2016)~~ [4]) with census information in a framework of optimized hot spot analysis~~.~~
Data source location	South of Mexico City
Data accessibility	Data are included in this paper

Value of the ~~data~~Data

- ~~The~~As the frontiers of urbanization are spaces ~~of~~with a high ~~conflict~~ potential ~~and~~for conflict, this new data and combination ~~type~~of data types present a methodology with the capacity for replication of studies in many cities.
- A combination of human (VGI) and remote sensing is relevant for a more reliable and validated analysis of urban growth.
- Due to the ~~falling~~decreasing costs of satellite imagery and the increasing availability of VGI, this type of analysis ~~shows a~~ has been proven to be cost-~~-~~effective ~~way~~ for future urban growth ~~investigation~~research.
- Other socio-~~-~~economic variables can be easily integrated ~~in~~into the analysis for further investigation.

Data

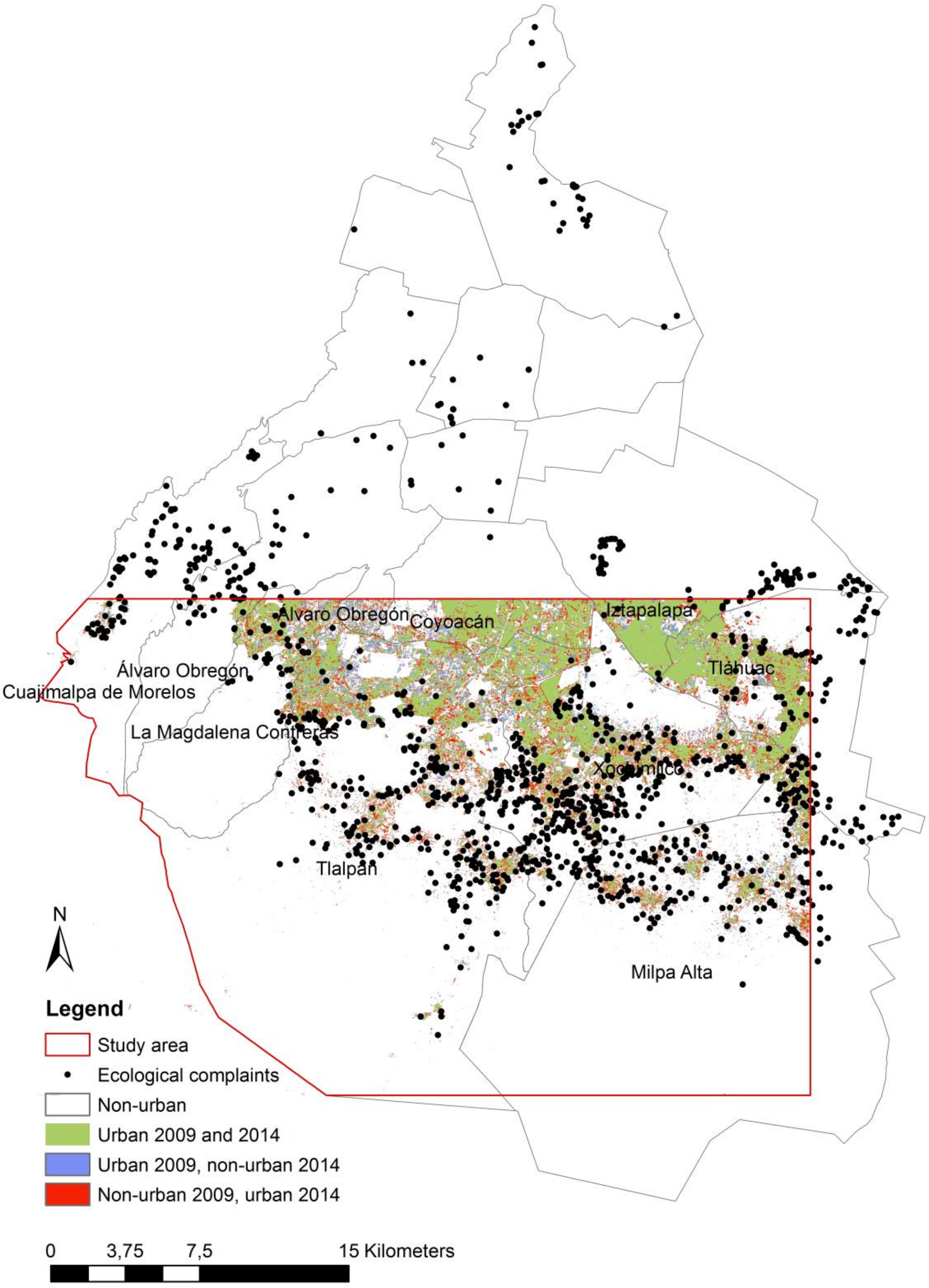

Figure 1: Data for the southern part of the Federal District in Mexico City: urban change, calculated from 5m remote sensing imagery of the RapidEye Science Archive (RESA) program, and ecological complaints (human sensing) from PAOT [around here]

For the urban change data, two tiles (IDs 1447913 and 1447914) of RapidEye 3A level products (5m per pixel) covering the south of Mexico City in November 2009 and August 2014 were classified using E-cognition~~ (Rodriguez Lopez et al. 2015).~~[5], which is a standard software for land use change analysis. Afterwards, ~~a~~we conducted change detection~~ was conducted.~~. The given urban change data ~~contains~~contain four classes: 0 = non-urban in 2009 and 2014; 1 = urban in 2009 and 2014; 2 = urban in 2009 but non-urban in 2014; and 3 = non-urban in 2009 but urban in 2014. The ecological complaints were reported between 2002 and 2013. ~~In the~~The data set~~,~~ includes 971 complaints concerning violations ~~in~~ of the status of the conservation area ~~are included~~ (see Figure 1~~). The~~ and related paper [4] for an explanation of "conservation area"). The smallest available basic geostatistical area (AGEB~~ data contains~~) contained a shape file with population data already included in the attributed table and a table containing economic characteristics for the Federal District of Mexico City. Important abbreviations for our analysis in the data set include: pop1 = total population and eco25_R ~~-~~= unemployment rate. ~~-~~A shape file of the study area is included (see Figure 1~~). Additionally,~~) as well as an ArcGIS toolbox containing the ~~model builder~~ModelBuilder for the analysis of the urban change data~~ is included. In~~. Figure 3 illustrates the model~~ is illustrated~~. The same workflow ~~is~~was added as python code.

Experimental Design, Materials, and Methods

~~The~~

We investigated the growth of ~~the~~ illegal settlements ~~in~~on the frontiers of urbanization in Mexico City ~~has been investigated~~ using a combination of human observation (VGI) and remote sensing (~~satellite imagery) (~~see Figure 2). A graphical abstract can be found in the associated publication ~~Rodriguez Lopez et al. 2016. This analysis was carried out to combine both databases in order to verify the validity and reliability of the data.~~[4]. In the following analysis, statistically significant urbanization hot spots ~~of human and remote sensing are~~were identified and investigated~~.~~ based on human and remote sensing. Integrating socio~~-~~-economic drivers ~~of~~from the census ~~(INEGI, 2010),~~[3], a statistical analysis of the causes ~~behind~~of the growth ~~of the~~in urbanization follows~~.~~ in future publications.

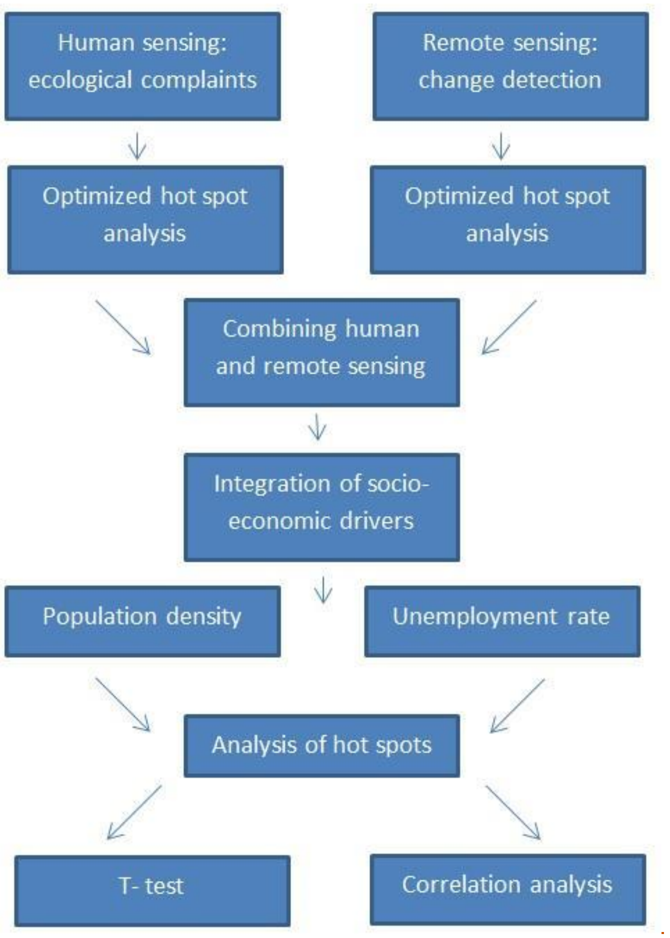

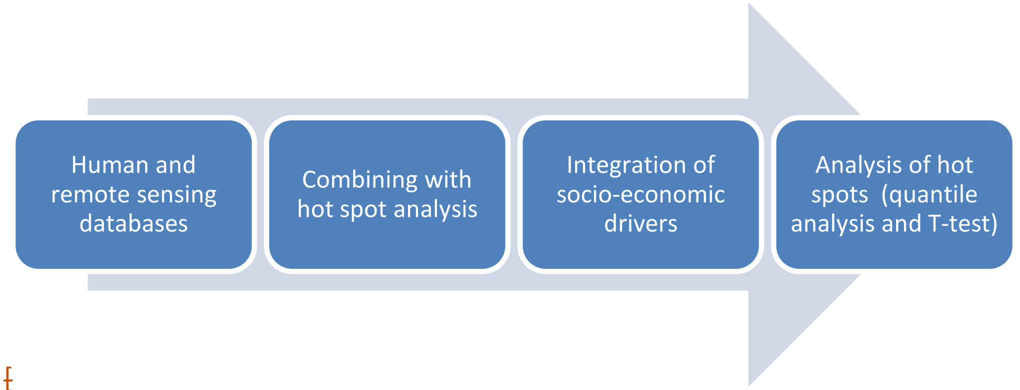

~~[~~

Figure 2: Overview of methods, combining human and remote sensing~~]~~ [around here]

~~In the following analysis~~ In order to compare data from both human and remote sensing~~ are investigated. For the comparison of both data sets~~, it ~~is~~was necessary to work with the same data type. Vector data ~~is favourable~~were favorable for most parts of the analysis. As the ecological complaints ~~are~~were already available as point data, the urbanization class from the change detection based on RapidEye imagery ~~has~~had to be extracted and converted to points. Each pixel ~~which is~~ classified as non-urban in 2009 and urban in 2014 ~~is~~was converted into one point. In the next step~~-~~, we conducted the optimized hot spot analysis ~~can be conducted~~ using ArcGIS 10.3. Within the analysis, statistically

significant hot and cold spots arewere identified usingwith the Getis-Ord Gi* statistic (Getis and Ord 1992). An[1]. The analysis of the cluster iswas based on the distance between each point (i.e.., every pixel in the remote sensing data or every complaint in the complaints database) and the nearest neighbour. Thisneighbor. In this analysis makes a comparison between, we compared the observation points and a random sample, obtaining a statistically significant classification of areas in various forms of clustering: hot and cold spots (Ord and Getis 1995).[2]. Polygons will bewere the spatial units of aggregation. The cell size of the remote sensing data iswas adapted to the cell size of the complaints data set, creating a grid with thea target resolution of 561m, before conducting the optimized hot spot analysis for the remote sensing data. was conducted. In the complaints data set, 561m generatesgenerated the best results for the autocorrelation test forof the automatic identification of hot and cold spots. While significant hot spots receivereceived a positive value, significant cold spots receivereceived a negative one. The significance lies atwas 90 percent% for values of +/- 1, at 95 percent% for values of +/- 2, and at 99 percent% for values of +/- 3. FeaturesA lower level of significance was assigned with a value of 0 are not significant. The optimized hot spot analysis is executed for the ecological complaints and for the remote sensing data. In Figure 3 an image of the model builder using ArcGIS 10.3 can be seen. It shows the steps for the analysis of remote sensing data and enables easy replication. To identify overlapping areas the hot spot maps are overlaid. Using the intersect tool, it is possible to identify the exact number of overlapping cells. To calculate how much of the observed urbanization by remote sensing is explained by human sensing, the number of cells belonging to hot spots with 99 percent confidence for both data sets is analysed (see Rodriguez Lopez et al. 2016)..

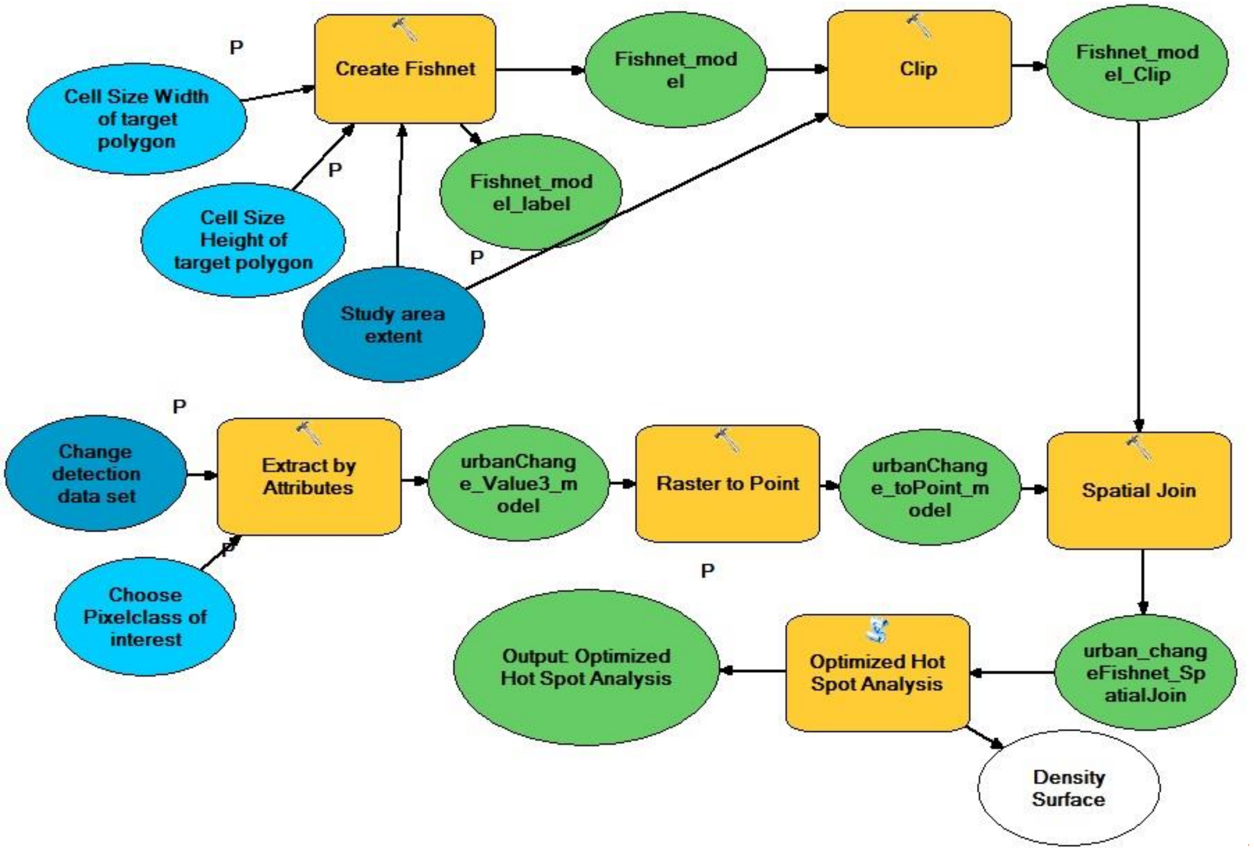

~~[Figure 3 Model builder representing the analysis of urban change data based on 5m remote sensing imagery to obtain statistically significant hot spots][around here]~~

An optimized hot spot analysis was executed for the ecological complaints and for the remote sensing data. Figure 3 shows an image of the ModelBuilder using ArcGIS 10.3, as well as the steps for the analysis of remote sensing data, which allows for easy replication. Data for the analysis and an ArcGIS toolbox containing the ModelBuilder are included in this paper. The ModelBuilder shows a standard representation of the analysis produced by ArcGIS. P indicates a model parameter, the blue and green ovals represent input and output variables, and yellow is a tool element; for a detailed description of the ArcGIS ModelBuilder's elements see the ESRI tutorial.[1] For the application, we added the data and toolbox to ArcGIS and we privileged here the original display of the ModelBuider to ease replication, although it is not a perfect figure (for example, line breaks in boxes). Figure 4 shows the interface of the ModelBuilder. To identify overlapping areas, we overlaid the hot spots maps. The intersect tool made it possible to identify the precise number of these overlapping cells. To calculate how much human sensing explained the observed urbanization by remote sensing, we analyzed the number of cells belonging to hot spots with 99% confidence for both data sets [4].

[1] Esri tutorial of the ModelBuilder can be found here: http://help.arcgis.com/en/arcgisdesktop/10.0/pdf/creating-tools-in-modelbuilder-tutorial.pdf (Accessed 20.12.2016)

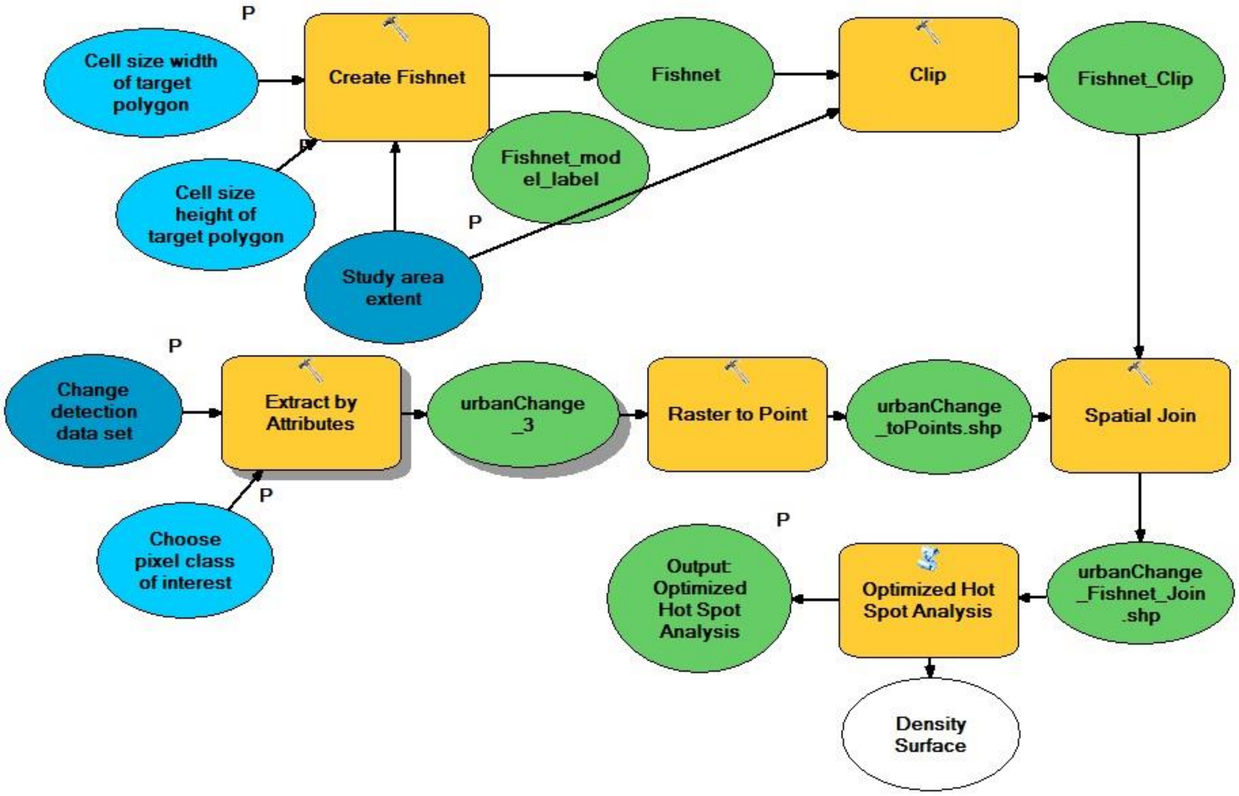

Figure 3: ModelBuilder representing the analysis of urban change data based on 5m remote sensing imagery (RESA program) to identify statistically significant hot spots [around here]

However, a key limitation of this approach ~~with~~using ArcGIS is the cost associated ~~to this licensed~~ with software licensing. The ~~python code~~Python script allows for a more transparent presentation of the analysis with this software. A file with the detailed code ~~is added here. It~~ has ~~to be taken into account~~ been added here. A particular note is that the ~~name~~names of the folders and paths should be ~~corrected~~adjusted to run this file. We recommend using a file geodatabase for data storage.

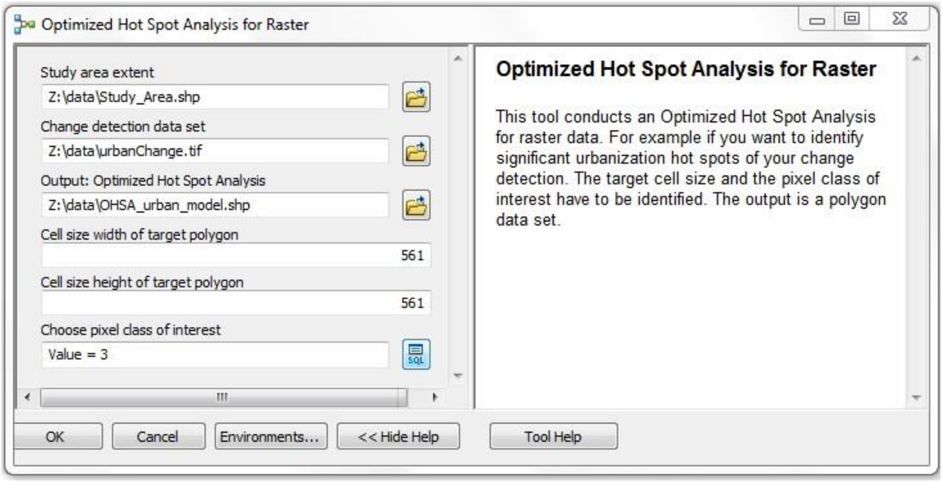

Figure 4: Interface of the ModelBuilder included in this paper (note: for application, all file paths need to be adapted)

For the integration of ~~socioeconomic~~socio-economic drivers, population and unemployment data ~~of~~from Mexico City, ~~the smallest available basic geostatistical area called 'AGEB', is~~AGEB data was used from the National Census 2010 ~~(National Census Bureau in Mexico, INEGI, 2010). Due to the high resolution of the geostatistical area, a higher resolution was chosen for this part of the analysis. The AGEB areas have a high variety of different sizes.~~[3]. The AGEB areas differed greatly in size. While the smallest area ~~is~~was 0.006 ~~-~~km^2, the biggest area ~~is~~was 7.25 km^2. The mean size ~~is~~was 0.4 km^2 with a standard deviation of 0.504 km^2. To minimize the loss of accuracy and ~~stay consistent within~~ ensure the consistency of our analysis, we chose a grid size of 0.143 km^2. To transfer the data to the target grid (378 m), we made a spatial join ~~is made~~ using the mean value as the merge rule and intersect as the match option. ~~In the next step, the data is classified in equal intervals and the number of cells within a hot spot belonging to the corresponding class is identified (see Table 2 and 3 in Rodriguez Lopez et al. 2016).~~ Although the fishnet ~~is~~was smaller, both the fishnet and AGEBs ~~are~~were comparable because the difference ~~is~~was acceptable, with a loss of accuracy. Both layers are shown in Figure ~~4~~5 to estimate the loss of accuracy.

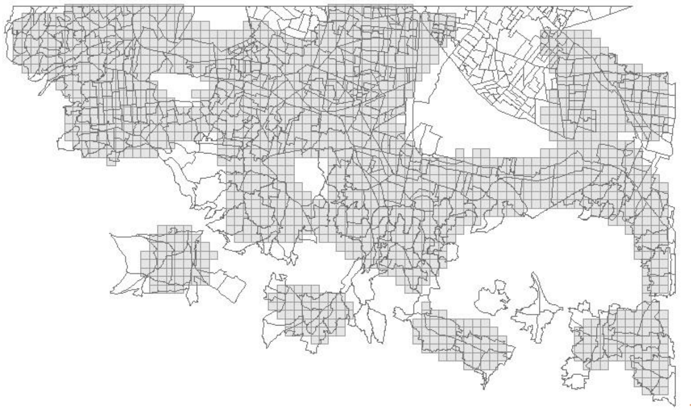

~~[~~

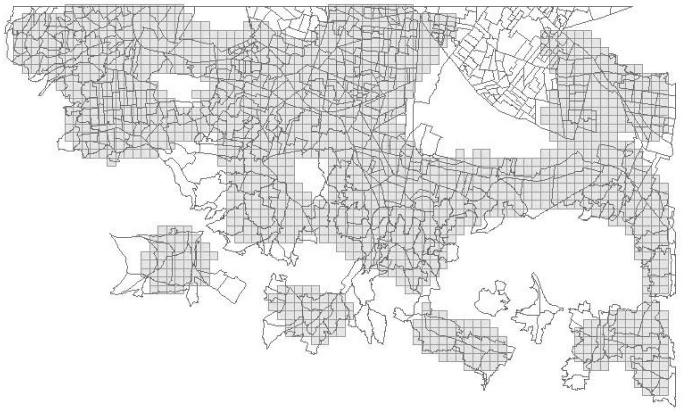

Figure ~~4~~5: Size distribution of AGEB and fishnet in the Federal District (overlay~~)][~~) [around here]

~~In order to explain the association between hot spots and population or socioeconomic variables, this article uses the Pearson correlation coefficient, which explains the degree and the direction of a linear association between two variables (see Rodriguez Lopez et al. 2016). Additionally, a t-test compares the difference in the means of hot spots and non-hot spots for the two variables (see Rodriguez Lopez et al. 2016).~~

Acknowledgements

This work was supported in ~~parts~~part by the Centre for a Sustainable University (KNU) and by the Cluster of Excellence ~~Cluster~~ "Integrated Climate System Analysis and Prediction" (CliSAP - EXC177) funded ~~by~~through the ~~Deutsche Forschungsgemeinschaft~~German Science Foundation (DFG). Underlying RapidEye data has been contributed on behalf of the German Aerospace Center through funding by the German Federal Ministry of Economy and Energy. Underlying RapidEye data has been contributed on behalf of the German Aerospace Center through funding by the German Federal Ministry of Economy and Energy.

References

Bimonte, S., Boucelma, O., Machabert, O., & Sellami, S. (2014). A new Spatial OLAP approach for the analysis of Volunteered Geographic Information. Computers, Environment and Urban Systems, 48, 111-123. doi:10.1016/j.compenvurbsys.2014.07.006.

Clark, M. L., & Aide, T. M. (2011). Virtual interpretation of Earth Web-Interface Tool (VIEW-IT) for collecting land-use/land-cover reference data. Remote Sensing, 3(3), 601-620. doi:10.3390/rs3030601.

Elwood, S. (2010). Geographic information science: Emerging research on the societal implications of the geospatial web. Progress in Human Geography, 34(3), 349-357. doi:10.1177/0309132509340711.

[1] Getis, A. & ,Ord, J.K. (1992). JK. The Analysis of Spatial Association by Use of Distance Statistics. Geographical Analysis,. 1992 24(3), :189-206. doi: 10.1111/j.1538-4632.1992.tb00261.x.

[2] Getis, A. &, Ord, J.K. (1995). JK. Local Spatial Autocorrelation Statistic: Distributional Issues and an Application. Geographical Analysis,. 1995 27(4), :286-306. doi:10.1111/j.1538-4632.1995.tb00912.x.

Goodchild, M. F., & Glennon, J. A. (2010). Crowdsourcing geographic information for disaster response: a research frontier. International Journal of Digital Earth, 3(3), 231-241. doi:10.1080/17538941003759255.

Hagenlocher, M., Lang, S., & Tiede, D. (2012). Integrated assessment of the environmental impact of an IDP camp in Sudan based on very high resolution multi-temporal satellite imagery. Remote Sensing of Environment 126, 27–38. doi:10.1016/j.rse.2012.08.010.

[3] INEGI. Estadísticas Censales a Escalas Geoelectorales. Principales resultados del Censo de Población y Vivienda 2010. (2012). http://gaia.inegi.org.mx/geoelectoral/doctos/FD_SECC_IFE.pdf.2012. http://gaia.inegi.org.mx/geoelectoral/doctos/FD_SECC_IFE.pdf. Accessed 17.12.2015.

[4] Rodriguez Lopez, J. M.,, Heider, K., &, Scheffran, J. (2016).Frontiers of Urbanization: Identifying and Explaining Urbanization Hot Spots in the South of Mexico City Using Human and Remote Sensing. Applied Geography, in press.. 2017 79C:1-10.

[5] Rodriguez Lopez, J. M.,, Rosso, P.,, Scheffran, J., & ,Delgado Ramos, G. C. (2015). Remote Sensing of Sustainable Rural-Urban Land Use in Mexico City: a Qualitative Analysis for Reliability and Validity. Interdisciplina,. 2015 3(7), :321-342.
