# Supplementary figures and images for: Human and remote sensing data to investigate the frontiers of urbanization in the south of Mexico City
Source: Data Brief. 2016 Dec 29;11:5–11. doi: 10.1016/j.dib.2016.12.049 (PMC5227549; doi:10.1016/j.dib.2016.12.049)

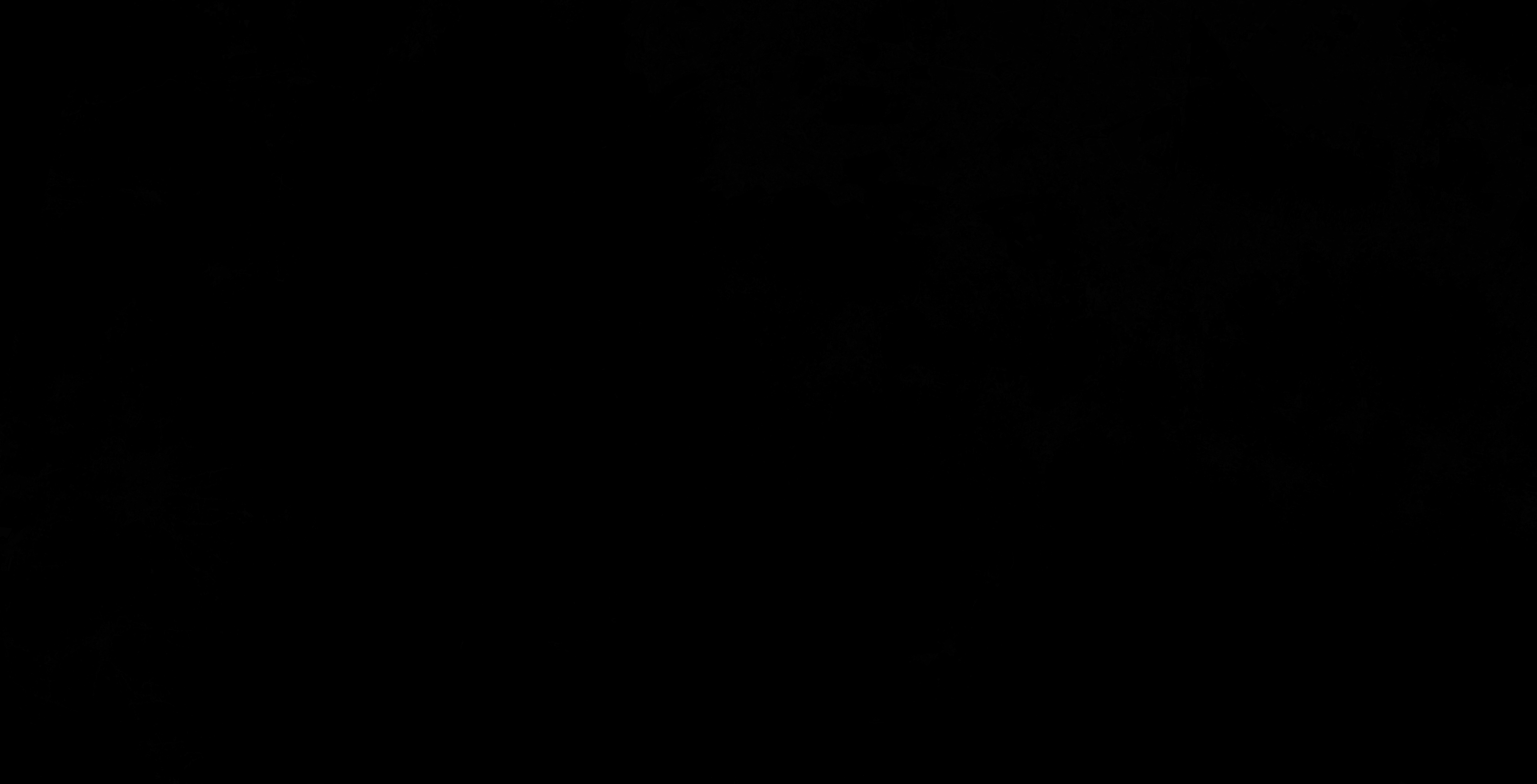

Supplement: Supplementary file 3 — Supplementary material [file mmc3.zip › Data_RodriguezHeiderScheffran/data/urbanChange.tif]

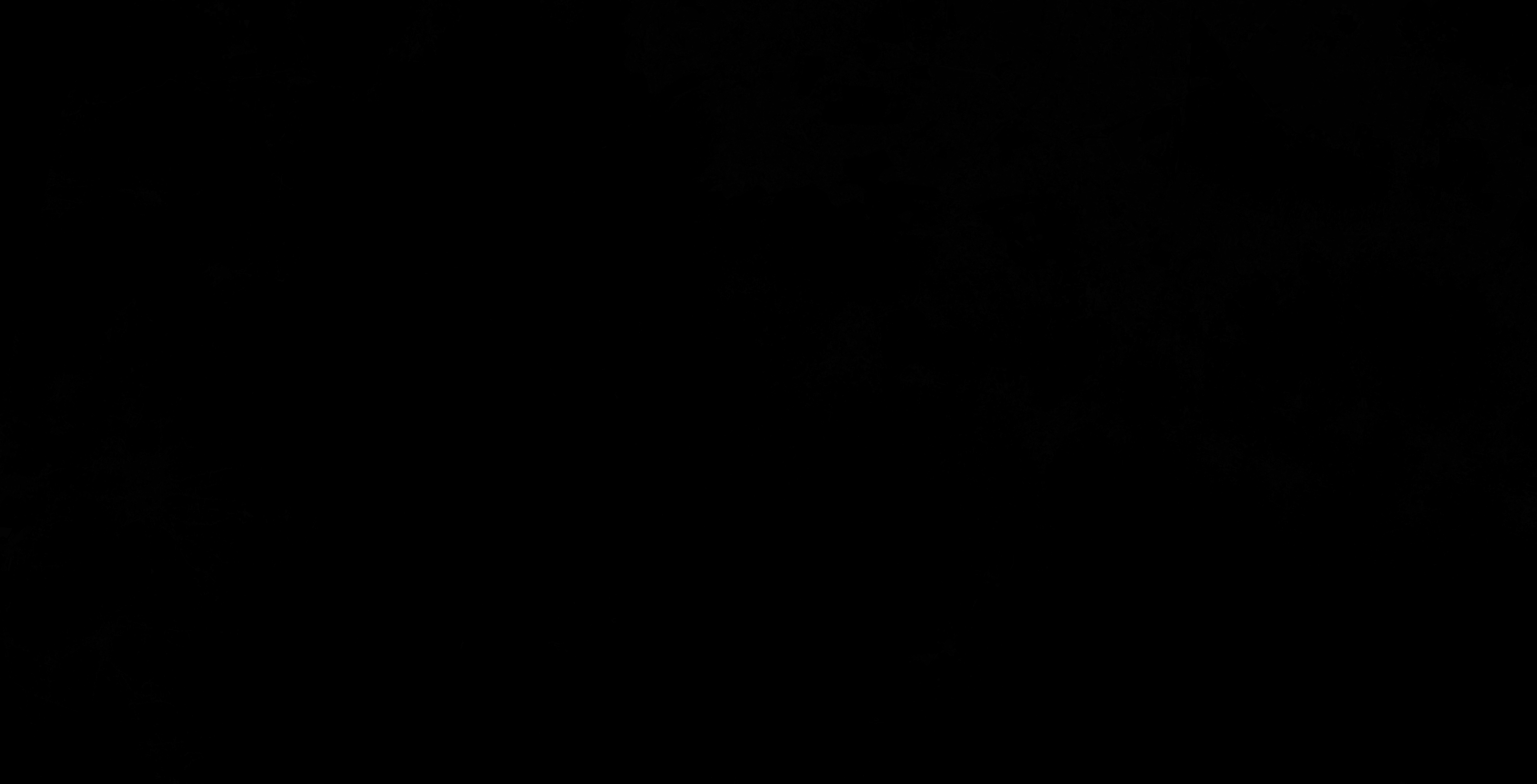

Supplement: Supplementary file 3 — Supplementary material [file mmc3.zip › Data_RodriguezHeiderScheffran/data/urbanChange.tif.ovr]
