# Supplementary material for: Human and remote sensing data to investigate the frontiers of urbanization in the south of Mexico City
Source: Data Brief. 2016 Dec 29;11:5–11. doi: 10.1016/j.dib.2016.12.049 (PMC5227549; doi:10.1016/j.dib.2016.12.049)
Supplement: Supplementary file 1 — Supplementary material [file mmc1.pdf]

Universität Hamburg
DER FORSCHUNG | DER LEHRE | DER BILDUNG

clisap

UHH · CliSAP Hamburg· Dr. Rodriguez Lopez
Grindelberg 5 · 20144 Hamburg

Marina Ostankovitch, PhD
Senior Scientific Editor
Data in Brief

Dr. Miguel Rodriguez Lopez
University of Hamburg
KlimaCampus Hamburg
Grindelberg 5 (R. 2009)
20144 Hamburg

Tel. +49 (0)40 42838-8619
Fax +49 (0)40 42838-6404
miguel.rodriguez@uni-hamburg.de

Date 21/12/2016

Dear Dr. Ostankovitch,

We wish to confirm that there are no known conflicts of interest associated with this publication and there has been no significant financial support for this work that could have influenced its outcome. We confirm that the manuscript has been read and approved by all named authors and that there are no other persons who satisfied the criteria for authorship but are not listed. We further confirm that the order of authors listed in the manuscript has been approved by all of us.

We confirm that we have given due consideration to the protection of intellectual property associated with this work and that there are no impediments to publication, including the timing of publication, with respect to intellectual property. In so doing we confirm that we have followed the regulations of our institutions concerning intellectual property.

We further confirm that any aspect of the work covered in this manuscript that has involved either experimental animals or human patients has been conducted with the ethical approval of all relevant bodies and that such approvals are acknowledged within the manuscript.

Yours sincerely,

Dr. Miguel Rodriguez Lopez on behalf of the authors
